# Candidaemia and antifungal therapy in a French University Hospital: rough trends over a decade and possible links

**DOI:** 10.1186/1471-2334-6-80

**Published:** 2006-05-02

**Authors:** Boualem Sendid, Angélique Cotteau, Nadine François, Annie D'Haveloose, Annie Standaert, Daniel Camus, Daniel Poulain

**Affiliations:** 1 Inserm, U799, Lille, F-59045 France;Laboratoire de Mycologie Fondamentale et Appliquée, Faculté de Médecine, Lille, F-59045, France; 2 Laboratoire de Parasitologie-Mycologie, CHRU, Lille, F-59045, France; 3 Département de Pharmacie, CHRU, Lille, F-59045 Lille, France

## Abstract

**Background:**

Evidence for an increased prevalence of candidaemia and for high associated mortality in the 1990s led to a number of different recommendations concerning the management of at risk patients as well as an increase in the availability and prescription of new antifungal agents. The aim of this study was to parallel in our hospital candidemia incidence with the nature of prescribed antifungal drugs between 1993 and 2003.

**Methods:**

During this 10-year period we reviewed all cases of candidemia, and collected all the data about annual consumption of prescribed antifungal drugs

**Results:**

Our centralised clinical mycology laboratory isolates and identifies all yeasts grown from blood cultures obtained from a 3300 bed teaching hospital. Between 1993 and 2003, 430 blood yeast isolates were identified. Examination of the trends in isolation revealed a clear decrease in number of yeast isolates recovered between 1995–2000, whereas the number of positive blood cultures in 2003 rose to 1993 levels. The relative prevalence of *Candida albicans *and *C. glabrata *was similar in 1993 and 2003 in contrast to the period 1995–2000 where an increased prevalence of *C. glabrata *was observed. When these quantitative and qualitative data were compared to the amount and type of antifungal agents prescribed during the same period (annual mean defined daily dose: 2662741; annual mean cost: 615629 €) a single correlation was found between the decrease in number of yeast isolates, the increased prevalence of *C. glabrata *and the high level of prescription of fluconazole at prophylactic doses between 1995–2000.

**Conclusion:**

Between 1993 and 2000, the number of cases of candidemia halved, with an increase of *C. glabrata *prevalence. These findings were probably linked to the use of Fluconazole prophylaxis. Although it is not possible to make any recommendations from this data the information is nevertheless interesting and may have considerable implications with the introduction of new antifungal drugs.

## Background

Bloodstream infections are a major cause of morbidity and mortality in developed countries. Recent statistics in the USA show that these infections are the tenth leading cause of death overall [1]. Their true incidence remains unknown, but it is estimated that around 250 000 cases occur annually in the USA alone [2]. In France, the prevalence of septicaemia in the hospital environment has been estimated to be around 0.4% [3]. Infections due to *Candida *species are an increasingly important complication in hospitalised patients [4-6]. North American and European surveillance studies have provided recommendations about the definition of risk factors and epidemiology of candidaemia [7,8]. Large scale epidemiological studies conducted in Europe [9] and the USA [10] show that *Candida *species are now the fourth most common cause of hospital-acquired bloodstream infection [11]. *Candida albicans *is the most common cause of candidaemia, and in general has remained susceptible *in vitro *to both polygenic drugs and fluconazole (FCZ) [12,13]. As the use of FCZ has increased, however, it has become more important to screen for azole resistance among bloodstream isolates or an increase in frequency of bloodstream infection due to species other than *C. albicans*, which have a higher incidence of *in vitro *azole resistance (e.g., *C. glabrata *and *C. krusei*) [14,15]. In this study, a longitudinal surveillance of bloodstream infections caused by *Candida *species was carried out in a 3300 bed teaching hospital during the 10-year period 1993–2003. The results were correlated with the use of FCZ over the same period.

## Methods

### Mycological investigations

A retrospective study of positive blood cultures for *Candida *species between 1993–2003 was carried out by the mycology service. This study allowed us to determine the number of cases of candidaemia annually; candidaemia was defined as one or more positive blood cultures for *Candida *species isolated from patients with clinical signs of infection. During this period three blood culture systems were used: Bio Argos (Sanofi Diagnostics Pasteur, Marnes-la-Coquette, France) and BactAlert 3D (Organon Teknika, France) were used successively by the bacteriology laboratory for the isolation of microorganisms in cases of septicaemia. When yeasts were isolated, the strain was transferred to the mycology laboratory for identification. In parallel, the Bactec 9050 system with aerobic mycosis IC/F medium (Becton Dickinson, USA) has been used by the mycology laboratory since November 1999. Mycosis IC/F medium, which is specifically adapted for the growth of fungi, significantly reduces the mean time for yeast detection(in particular *C. albicans *and *C. glabrata*) [16].

For all blood culture systems, the blood culture flasks were mixed with 10 ml of blood and then incubated at 37°C for 7 days. A control for microbial growth was carried out automatically.

### Identification of strains

Positive samples were examined directly after staining with Giemsa or toluidine blue and subculture on Sabouraud's agar containing gentamycin (40 mg/L). All strains isolated were identified using the germ tube test, chlamydospore production and API 32C system (Bio-Mérieux, Marcy l'Etoile, France). Since 1997, all strains have also been subcultured on chromogenic medium CHROMagar, which allows the presumptive identification of some *Candida *species. The germ tube test has been replaced with Bichrolatex *albicans*, a rapid immunological test for the identification of *C. albicans*.

### Pharmaco-economic investigations

The annual consumption of antifungal drugs during the same period was determined from pharmacy data (TAGE, GEF, and Mc Kesson softwares). The annual consumption of a drug takes into account all hospital services using the drug, with any eventual returns of the product deducted. This consumption is expressed as total milligrams of active drug used. The antifungal drugs included in the evaluation were indicated for either prophylaxis or curative treatment of candidaemia: 5-fluorocytosine (5FC; Ancotil^®^), amphotericin B (AMB; Fungizone^®^), amphotericin B lipid complex (ABLC; Abelcet^®^), liposomal amphotericin B (AMBD; AmBisome^®^), caspofungin (CAS; Cancidas^®^), voriconazole (VCZ; Vfend^®^) and fluconazole (FCZ; Triflucan^®^). The data collected included antifungal use and the number of cases of candidaemia observed during the same period.

## Results

### Distribution of candidaemia cases over the study period

**Figure 1:**
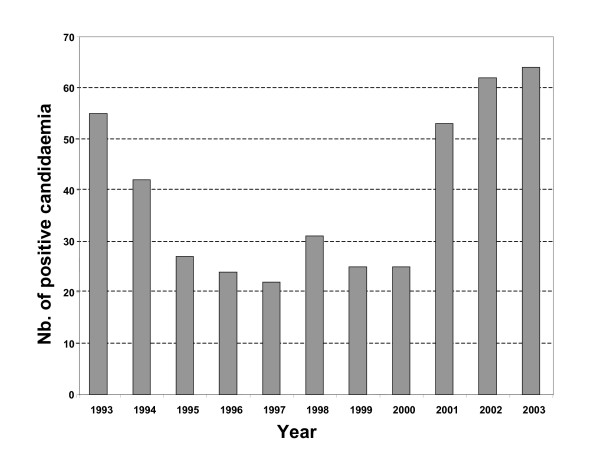
Number of episodes of candidaemia recorded during 1993–2003 in a 3300 bed French university hospital.

**Figure 2:**
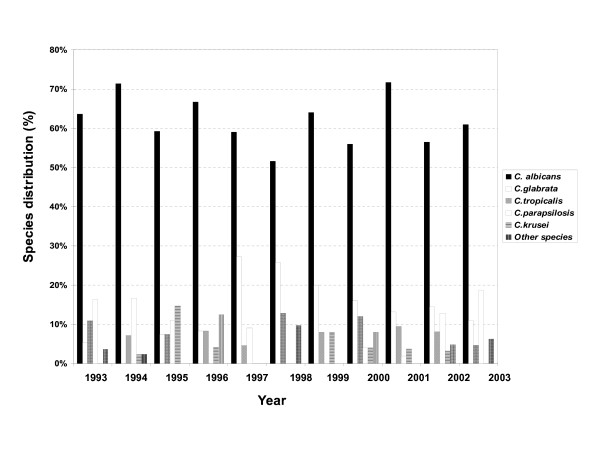
Distribution of *Candida *species as bloodstream isolates by year (1993–2003).

**Figure 3:**
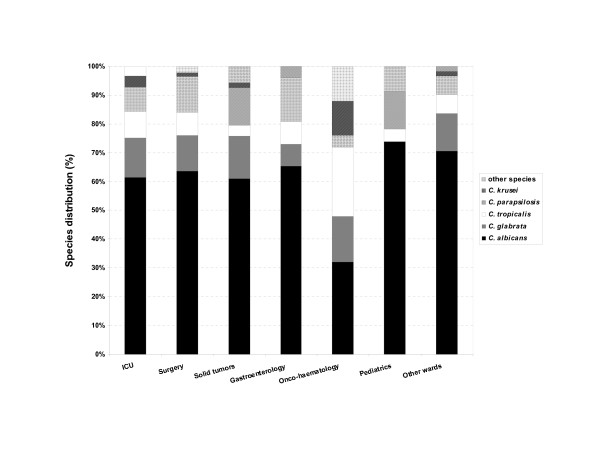
Distribution (percentage) of the most frequently isolated *Candida *species according to the underlying condition.

During the period 1993–2003, an average of 45762 blood cultures were analysed annually by the bacteriology laboratory and an average of 659 by the mycology laboratory. Among these, 430 (1/1000; 0.1%) were positive for *Candida *species compared with 11% for bacteria. The cases of candidaemia originated most frequently from the intensive care service (36%), surgical unit (20%), cancer unit (13%), gastroenterolgy (6%), haematology (6%) and paediatric service (5%). The other services (burns, geriatrics, pneumology, nephrology, internal medicine, etc.) represented only 14% of isolates overall. The annual incidence of positive blood cultures is shown in Figure 1. Between 1995 and 2001, a decrease in number of cases of candidaemia was observed. In general, *C. albicans *was the predominant species making up 61.5% of isolates. Conversely, a significant variation was observed for non-*albicans *species, with *C. glabrata *predominating between 1995 and 2001, with a peak incidence of 27% in 1997 (Figure 2). Outside this period, *C. parapsilosis *and *C. tropicalis *were the most frequent species isolated after *C. albicans. C. parapsilosis *was the predominant non-*albicans *species before 1995 (with a mean incidence of 14.7%), and after 2001. The mean incidence of *C. tropicalis *was 9%, with a peak of 13% in 1998. Little variation was observed for *C. krusei *which remained below 4%. The other species included *C. lusitaniae *(n = 3), *C. kefyr *(n = 2), *C. guilliermondii *(n = 2) and *C. norvegensis *(n = 1). The evolution and proportion of different *Candida *species in relation to the main hospital services caring for patients at risk of systemic candidosis is shown in Figure 3. *C. albicans *represented more than 60% of isolates recovered from the different services except for oncology/haematology where *C. albicans *represented only 32% of isolates. This reduction in isolation of *C. albicans *was associated with an increase in isolation of *C. tropicalis *(24%), *C. glabrata *(16%) and *C. krusei *(12%). No isolates of *C. glabrata *were recovered from the paediatric service. Although the number of strains isolated was small (n = 12), no case of candidaemia caused by *C. krusei *was observed in paediatrics or gastroenterology.

### Variation in consumption of antifungal drugs

Antifungal use in milligrams between 1993–2003 was converted to the defined daily dose (DDD) to enable interpretation. The DDD for the different drugs was: 70 mg for AMB, 210 mg for AMBD, 350 mg for ABLC, 400 mg for FCZ, 10 000 mg for 5FC, 50 mg for CAS and 400 mg for VCZ.

**Figure 4:**
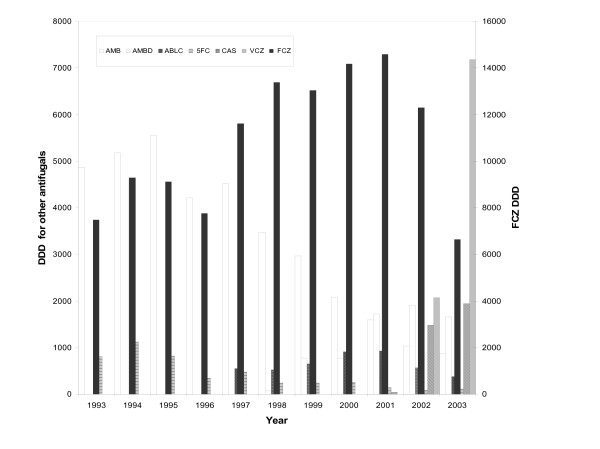
Annual consumption of systemic antifungal agents (daily doses) between 1993–2003. AMB: amphotericin B; AMBD; ambisome; ABLC: abelcet; 5FC: 5-fluorocytosine; CAS: caspofungin; VCZ: voriconazole; FCZ: fluconazole.

Figure 4 shows that different formulations of AMB were used constantly (mean DDD 5102), with a progressive decrease in AMB use after 1999 in favour of lipid formulations (ABLC and AMBD). ABLC has been used since 1997 with a constant mean annual consumption of 647 DDD. AMBD has been used since 1998 with a considerable increase in use documented since 1999. Low consumption of 5FC was noted over the whole study period (mean DDD: 425). Use of this drug, often as combined therapy, was significant until 1995.

Evaluation of FCZ use revealed a net increase since 1997. The mean DDD for the period 1993–1996 was 8412 compared with a mean DDD of 13184 between 1997–2002. The recent introduction of VCZ (2002) and CAS (2001) has increased the number of drugs available to treat candidaemia. The large increase in use of VCZ seems to correlate with a reduction in FCZ use in 2003 (Figure 4). CAS consumption is currently equivalent to that of AMBD.

### Variation in consumption of fluconazole related to candidaemia

**Figure 5:**
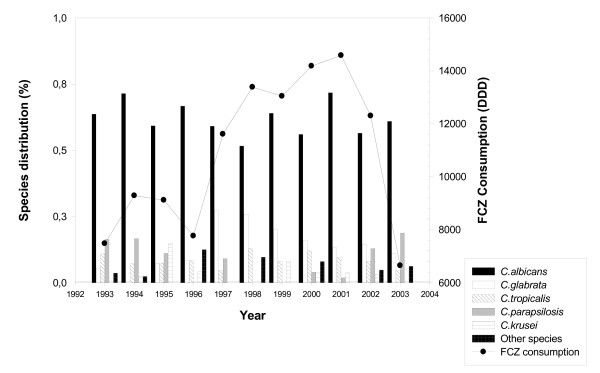
Evolution of isolation of *Candida *species in relation to fluconazole consumption between 1993–2003.

If FCZ consumption and profile of *Candida *species isolated from blood cultures are superimposed it can be seen that the increase in FCZ use correlates closely with a change in profile of non-*albicans *species isolated with *C. glabrata *predominating (Figure 5).

**Figure 6:**
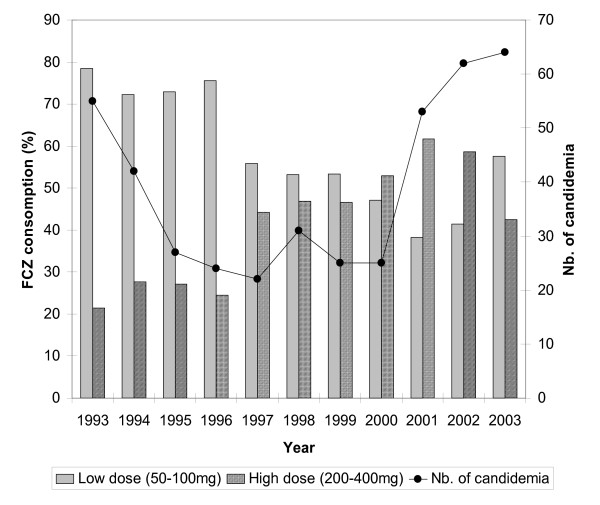
Annual consumption of low- and high-dose fluconazole in relation to the number of episodes of candidaemia between 1993–2003.

Analysis of FCZ consumption during the study period reveals that two types of prescription were possible: low-dose (dose between 50–100 mg) and high-dose therapy (dose >200 mg) (Figure 6). A reduction in prescription of lower doses was observed after 1997, coinciding with a 40% reduction in number of cases of candidaemia compared to 1993 and the emergence of *C. glabrata *as a significant cause of infection (27%) (Figure 6). An inversion in prescription of low- versus high-dose FCZ was observed after 2000. During the period 1998–2000, the ratio of the two doses was nearly 1. The relative peak in percentage of low-dose FCZ used in 2003 corresponded to a progressive reduction in high-dose FCZ in favour of VCZ.

## Discussion

The incidence of nosocomial fungal infections has continued to rise over the past two decades in parallel with advances in medical and surgical procedures. Bone marrow and solid organ transplant procedures, surgery and medical intensive care have largely increased the number of profoundly immunosuppressed patients at high risk of opportunistic infection [8,11]. Invasive fungal infections are usually severe in these patients and are difficult to diagnose and treat [17]. A significant proportion of these patients do not die from their underlying pathology but die of infectious complications often related to a deep fungal infection. *Candida *species are among the most frequently isolated agents of invasive fungal infection [18], and account for 8–10% of hospital-acquired cases of septicaemia [15]. In spite of recent progress in antifungal chemotherapy, the mortality rate from these infections continues to rise [19]. Their economic impact has also been recognised, linked largely to the empirical use of systemic antifungal drugs and a prolongation of hospital stay (on average 30 days), which is necessary for optimal treatment and control of candidaemia [20,21]. This overspend is estimated at 40.000 $ per patient in some American intensive care units [21].

Although the procedure suffers from a lack of sensitivity, blood culture remains an important tool in the diagnosis of invasive candidosis [22]. According to recommendations from some experts and learned societies, a single positive blood culture is sufficient to make a diagnosis of invasive candidosis and justifies the initiation of antifungal treatment [23]. In these conditions, epidemiological studies on the number and type of *Candida *species isolated from blood cultures are an important prerequisite to developing effective antifungal strategies.

The current report presents the results of a retrospective study of cases of candidaemia during the last decade (1993–2003), in a French university hospital. The study did not include patients with HIV, who were cared for in an infectious diseases service not attached to our hospital.

Analysis of the data obtained demonstrated that the number of cases of candidaemia halved between 1993 and 2000. This trend was probably linked to the use of FCZ prophylaxis. The results also demonstrated that *C. albicans *was the most frequently isolated species, comprising around 60% of isolates, irrespective of the medical specialty. These results differ from recent American studies, which reported a lower isolation rate for *C. albicans *of around 50% [10]. In our study, a single exception was noted in the oncology/haematology units where *C. albicans *represented less than 35% of isolates, while the majority of isolates were species such as *C. glabrata, C. tropicalis *and *C. krusei *which are less sensitive or resistant to azoles. This change in epidemiology of candidaemia in oncology/haematology confirms the results reported in a multicentre European study where *C. albicans *represented only 34.6% of isolates [9]. This emergence of non-*albicans *species is probably linked to FCZ prophylaxis, which is carried out mainly during periods of profound neutropenia, particularly after allogeneic bone marrow transplantation [24].

One notable finding from this investigation is the emergence of *C. glabrata *since 1996 (Figure 2). Analysis of the use of systemic antifungal drugs reveals an exponential increase in FCZ use between 1993 and 2002, with an increased use of low doses of this drug (generally used for prophylaxis) until 1996. The emergence of *C. glabrata *appears to be linked to the use of low-dose FCZ before 1997. Several reports have demonstrated that the use of sub-optimal doses of FCZ (<400 mg) can increase the frequency of *C. glabrata *as a cause of candidaemia in hospitalised patients [25,26]. It is also important to note that the prominent use of high-dose FCZ was accompanied by a progressive reduction in isolation of *C. glabrata*, which fell to 10% of isolates in 2003. This reduction continued with the introduction of VCZ in 2002, leading to a significant reduction in use of high-dose FCZ (Figure 6). The low use of 5FC underlines the rarity of neuro-meningeal mycoses in our hospital. The introduction of lipid formulations of AMB has changed the use of this drug in patients likely to develop renal failure. In particular, AMBD is used widely as it has fewer side-effects and a broader range of indications than ABLC [27].

Although several mechanisms of resistance to azoles (FCZ, VCZ, ITZ) have been described in *Candida*, resulting in overexpression of CDR1/CDR2 or MDR1 genes, or mutation in the ERG11 gene [28], the use of FCZ remains justified. In effect, the proportion of strains of *Candida *species that are resistant to FCZ remains low (<10%). Among these, the percentage of *C. glabrata *strains varies from 0–23% according to the country [15,29]. Pressure for the selection of azole-resistant species has been reported by several authors [28], resulting in the introduction of recommendations for good use of antifungal drugs [18]. As far as FCZ is concerned, it has been recommended that patients are treated with curative doses of at least 400 mg/day, and that patients are stratified justifying antifungal therapy.

A reference system based on diagnostic strategies including clinical risk factors, scores for specific signs, radiological data and investigations based on culture of fungal pathogens has recently been set up. Taking into account the local ecology, the use of FCZ remains justified not just as prophylaxis but also as curative treatment for invasive candidosis.

The recommendations made distinguish two situations, prophylactic treatment in patients at high risk of invasive fungal infection, and treatment of clinically and mycologically documented invasive candidosis. The introduction of this reference system has resulted in a 13% reduction in cost of systemic antifungal agents (2.476.438 € in 2004 *versus *2.814.617 € in 2003). Nevertheless, it does not appear to have had a significant impact on the incidence of candidaemia, which continues to rise (70 in 2004 vs. 38 in 1993). These data suggest that better diagnostic monitoring is required to allow earlier identification of patients at high risk of fungal infection. In addition to reference tests such as blood culture and histology this approach should include other biological markers (inflammatory markers, determination of fungal colonisation, antigens, antibodies, nucleic acid, metabolites) whose circulation often precedes the isolation of fungi from a sterile site. Combination of these tests should improve the level of certainty of a fungal infection and the early initiation of antifungal treatment, resulting in a better prognosis. The efficacy of such measures should be evaluated regularly and adapted to the local ecology taking into account the increase in indications for the different drugs, the introduction of new antifungal agents and progress in diagnostic strategies.

## Conclusion

The present study reveals that the number of cases of candidaemia in our hospital fell by more than 50% in 1997 compared with 1993 and 2003. Analysis of antifungal use revealed that this period corresponded to a peak in prescription of oral forms of FCZ for prophylactic use. At this time the number of isolates of *C. glabrata *was almost 50% of the number of isolates of *C. albicans*. As *C. glabrata *is known to be resistant to FCZ, it is likely that this correlation is not due to chance.

## Competing interests

The author(s) declare that they have no competing interests.

## Authors' contributions

BS and DP conceived the study, directed its design and execution, and drafted the manuscript.

NF performed the mycological analysis, collected the data and participated to their interpretation. AC and AD collected and provided data on consumption of antifungal drugs, and participated to the redaction of the manuscript. AS participated in data collection and analysis and helped to draft the manuscript. DC managed the activity of the Parasitology-Mycology Clinical Laboratory and the laboratory staff. All authors read and approved the final manuscript.

## Pre-publication history

The pre-publication history for this paper can be accessed here:


